# Decomposition rate and biochemical fate of carbon from natural polymers and microplastics in boreal lakes

**DOI:** 10.3389/fmicb.2022.1041242

**Published:** 2022-11-08

**Authors:** Jussi S. Vesamäki, Riitta Nissinen, Martin J. Kainz, Matthias Pilecky, Marja Tiirola, Sami J. Taipale

**Affiliations:** ^1^Department of Biological and Environmental Science, Nanoscience Center, University of Jyväskylä, Jyväskylä, Finland; ^2^WasserCluster Lunz—Biological Station, Donau-Universität Krems, Lunz am See, Austria

**Keywords:** decomposition, microplastic, polymer, mineralization, Burkholderiaceae

## Abstract

Microbial mineralization of organic compounds is essential for carbon recycling in food webs. Microbes can decompose terrestrial recalcitrant and semi-recalcitrant polymers such as lignin and cellulose, which are precursors for humus formation. In addition to naturally occurring recalcitrant substrates, microplastics have been found in various aquatic environments. However, microbial utilization of lignin, hemicellulose, and microplastics as carbon sources in freshwaters and their biochemical fate and mineralization rate in freshwaters is poorly understood. To fill this knowledge gap, we investigated the biochemical fate and mineralization rates of several natural and synthetic polymer-derived carbon in clear and humic lake waters. We used stable isotope analysis to unravel the decomposition processes of different ^13^C-labeled substrates [polyethylene, polypropylene, polystyrene, lignin/hemicellulose, and leaves (*Fagus sylvatica*)]. We also used compound-specific isotope analysis and molecular biology to identify microbes associated with used substrates. Leaves and hemicellulose were rapidly decomposed compared to microplastics which were degraded slowly or below detection level. Furthermore, aromatic polystyrene was decomposed faster than aliphatic polyethylene and polypropylene. The major biochemical fate of decomposed substrate carbon was in microbial biomass. Bacteria were the main decomposers of all studied substrates, whereas fungal contribution was poor. Bacteria from the family Burkholderiaceae were identified as potential leaf and polystyrene decomposers, whereas polypropylene and polyethylene were not decomposed.

## Introduction

Microbial decomposition of organic compounds is essential for carbon recycling in food webs. In addition to autochthonous carbon sources, freshwaters receive terrestrial loadings whose microbial decomposition processes are poorly understood. Terrestrial-derived organic carbon includes labile carbon, such as carbohydrates, polysaccharides, and cellulose, which are quickly utilized by microbes. In addition to these, terrestrial loadings also include more recalcitrant polymers, e.g., lignin and cellulose, both precursors for humus formation (Danise et al., 2018) and are found in high concentrations in boreal humic, brown-water lakes (Hessen and Tranvik, 1998). Clear-water lakes [defined often as DOC <10 mg/L (Kortelainen, 1993)] typically are rich in labile organic carbon sources, which are utilized efficiently by microbes (Bręk-Laitinen et al., 2012), whereas humic lakes have more recalcitrant carbon compounds (Hessen and Tranvik, 1998). It is thus plausible to assume that humic freshwater microbes are highly adapted to utilize diverse carbon sources and have developed a vast arsenal of biochemical weathering agents to use recalcitrant carbon sources. To date, microbial decomposition of lignin has been studied in soils even at the molecular level [e.g., (Pathan et al., 2017; Bhatnagar et al., 2018)] and sediments [e.g., (Song et al., 2019; Yang et al., 2021)], but their decomposition and the role of different microbial groups in the decomposition process in freshwaters are weakly understood.

In addition to natural polymers, microplastics have been found in several environments worldwide (Shahul Hamid et al., 2018), including lentic (Di Pippo et al., 2020; Uurasjärvi et al., 2020) and lotic (Crew et al., 2020; Mora-Teddy and Matthaei, 2020) freshwaters. Microplastic pollution has become an increasing environmental concern worldwide due to increasing plastic pollution in oceans (Jambeck et al., 2015) and freshwaters (Hurley et al., 2018). However, their mineralization rate and biochemical fate in freshwater systems remain poorly investigated (Anderson et al., 2016; Taipale et al., 2019). Until now, microplastic degradation studies have focused on microbes colonizing the surface of microplastic (e.g., Debroas et al., 2017) and pure culture testing (e.g., Yoshida et al., 2016). Currently used methods include imaging methods such as scanning electron microscopy (SEM) imaging, which does not give any information about the biochemical fate of plastic-derived carbon.

Bacterial diversity increases with microplastic surface roughness but does not vary among different plastic types (Di Pippo et al., 2020). Although particle size has not been found to significantly affect bacterial community composition between smaller and larger microplastic particles (Frère et al., 2018), bacterial diversity has been higher on larger mesoplastic particles than on microplastics and lowest on PS particles (Debroas et al., 2017). However, plastic’s physical properties and chemical composition play an important role in the decomposition process. Microbes can attach more easily to heteroatomic microplastics (Debroas et al., 2017) like nylon and polyvinyl. For example, freshwater fungi that grew on plastic particles were found to be able to degrade aromatic and nitrogen-involving polyurethane but not aliphatic polyethylene (Brunner et al., 2018). In contrast to microplastics, natural carbon sources can involve a diverse mix of easily utilizable carbon sources such as carbohydrates and heteroatomic compounds and more recalcitrant carbon sources such as benzene rings. For example, lignin and recalcitrant humic substances are mineralized slowly in humic waters (Vähätalo et al., 1999) whereas microbial glucose mineralization is fast (Schneckenberger et al., 2008).

Microbes can assimilate polymer carbon by extracellular enzymatic degradation or by endocytosis (Sánchez, 2020; Yuan et al., 2020; Liu L. et al., 2021). In addition, microbes can break chemical bonds of a polymer by enzymatic hydrolysis, and these oligo-, di- or monomers can be further processed intracellularly and mineralized to CO_2_ or CH_4_ or used for anabolic processes (Yuan et al., 2020; Du et al., 2021; Liu X. et al., 2021). Both mineralization rate and the biochemical fate of polymer carbon into inorganic carbon *via* respiration (CO_2_) and microbial biomass can be studied using ^13^C-labelled substrates and stable isotope analysis (Steffens et al., 2015; Yang et al., 2015; Taipale et al., 2019). Measuring the assimilation of ^13^C into phospholipid fatty acid (PLFA) biomarkers by compound-specific isotope analysis (CSIA) enables the tracking of the carbon cycle and decomposition pathways (Twining et al., 2020). Furthermore, PLFA-biomarkers and CSIA can be combined with microbial community analysis to identify microbial taxa responsible for decomposition (Taipale et al., 2019). However, only a few studies (e.g., Yang et al., 2015; Taipale et al., 2019; Liu L. et al., 2021) have used ^13^C-labeled microplastics to study microbe-driven plastic degradation, despite its high sensitivity and usefulness as a tool to study the decomposition of highly recalcitrant and slowly degraded materials.

In this study, we compared decomposition rate and pathways, and the biochemical fate of carbon from different natural polymers and microplastics using ^13^C-labeled materials to gain a deeper understanding of microbial decomposition processes in freshwaters. We selected deciduous tree leaves (*Fagus sylvatica*) and lignin-hemicellulose for natural carbon sources and polyethylene (PE), polypropylene (PP), and polystyrene (PS) for microplastics. We compared their decomposition rates and biochemical pathways in clear-water lake water containing labile organic carbon (Bręk-Laitinen et al., 2012) and humic lake water, where microplastic mineralization was observed in a previous study (Taipale et al., 2019). The following hypotheses were tested: (1) decomposition rate is faster in humic lake water than in clear lake water for microplastic polymers and lignin, whereas decomposition rate was assumed to be similar for leaves in both lake types, (2) decomposition is faster for aromatic polystyrene than for aliphatic polyethylene or polypropylene, (3) carbon from decomposed substrate is mainly utilized to build biomass whereas smaller proportion is respired, and (4) bacteria are primary decomposers for easily degradable carbon sources (lignin-hemicellulose and leaves), whereas fungi play a more important role in the decomposition of plastics.

## Materials and methods

### Sampling sites, experiment preparation, and experimental setup

**Figure 1:**
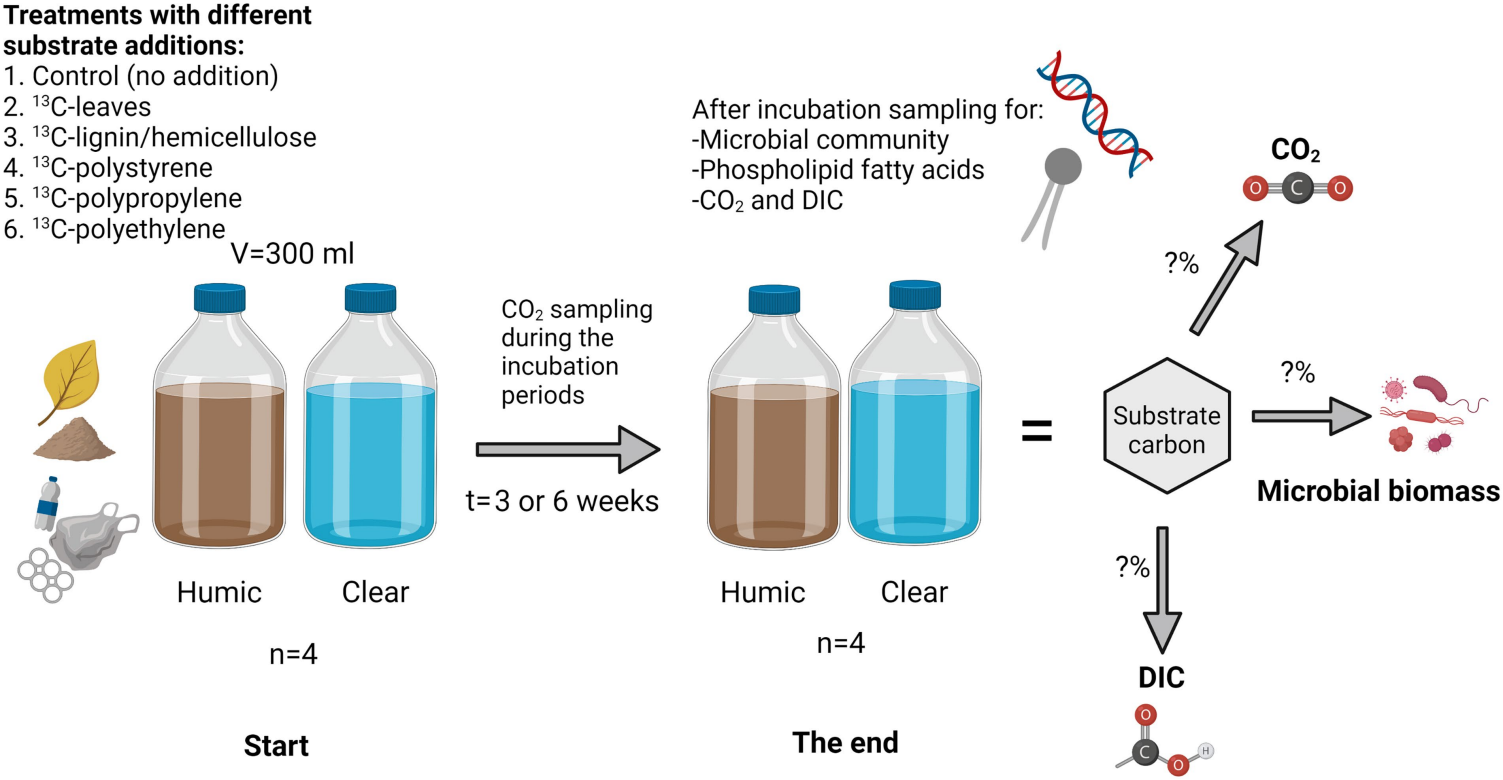
Experimental setup. Various carbon sources (1-6) were incubated in clear and humic lake water after which the transfer of carbon into microbial biomass and inorganic carbon was studied using stable isotope analyses. Microbial community analyses and compound-specific isotope analysis were used to identify microbial taxa involved in the decomposition processes.

Waters were collected from highly humic lake Nimetön [Evo, Finland 61°22′81″N, 25°19′23″E; DOC = 22.60 ± 0.88 mg/L] and from clear water lake Vesijärvi (Lahti, Finland, (61°02′42″N, 25°35′10″E; DOC = 5.19 ± 0.05 mg/L) in July 2020 for microplastic and lignin-hemicellulose treatments and in September 2020 for leaf treatments. Collected waters were filtrated through a 3 μM pore size filter to remove bacterivores and preincubated at 18°C for 3 weeks before the start of the experiment to let microbes consume most of the easily available carbon sources. After the preincubation, 300 mL of water was poured into a 540 mL glass bottle and 4 mg C of ^13^C-substrate (PE (Poly(ethylene-^13^C_2_) Sigma-Aldrich, 99 atom% ^13^C, United States); PP (Poly(propylene-1-^13^C) Sigma-Aldrich, 99 atom% ^13^C, United States); PS (Poly(styrene-α-^13^C) Sigma-Aldrich, 99 atom% ^13^C, United States); lignin-hemicellulose (U-13C lignin organosolv from wheat (*Triticum aestivum*), IsoLife bv 97 atom% ^13^C, Netherlands); NLD Hygroscopic); leaves (P-^13^C Beech leaf (*Fagus sylvatica*) 13.4 atom% ^13^C, IsoLife bv, Netherlands) was added. Used lignin-hemicellulose was composed approximately 80% of lignin and 20% of carbohydrates, including mostly hemicellulose (van Erven et al., 2017). Control treatments had no substrate addition. Lake waters with added substrates were incubated at 17–18°C in closed glass bottles for three (leaves), or six (lignin-hemicellulose, microplastics, and controls without any substrate addition) week(s) (Figure 1). Bottles were daily shaken during the experiment. Four replicates were made for each treatment.

### Mineralization

Gas samples were taken weekly from the air phase of a bottle. 5 mL of gas sample was transferred into an air-free Exetainer® tube. At the end of the experiment, ^13^C-DIC was analyzed by taking 5 mL of water into a He-flushed Exetainer® tube and adding 200 μL of 85% H_3_PO_4 (Taipale and Sonninen,__2009__)_. Water samples were mixed by a vortex and 5 mL of the gas phase was taken from the Exetainer® tube into a new tube. The gaseous DIC samples were further processed and analyzed identically to CO_2_ samples. The amount of CO_2_ in the sample was determined by an Agilent 7890B gas chromatograph (Agilent Technologies, Palo Alto, CA, United States). After the quantification of CO_2_ in sample tubes, δ^13^C values of CO_2_ and DIC were analyzed using an Isoprime TraceGas pre-concentrator unit connected to an Isoprime IRMS (Isoprime100 IRMS, Elementar UK Ltd., Cheadle, UK) at the University of Jyväskylä, Finland. δ^13^C values were drift corrected and two-point calibrated based on external standards. Mineralization rate calculations are presented in Supplementary material.

### Analysis of ^13^C-PLFAs by GC–MS, *CF*-IRMS, and compound-specific isotopes

At the end of the experiment, water was filtered through a preweighted filter (Whatman™ cellulose nitrate filters, pore size 0.2 μM, diameter 47 mm) and stored at −80°C. After that, filters were freeze-dried, weighed, and transferred into a Kimax® tube. 3 mL of chloroform-methanol (2:1) and 750 μL distilled water and internal standards C19:0 and C23:0 (0.4997 mg/mL and 0.5044 mg/mL, respectively) were added into a sample tube. Tubes were sonicated for 10 min and then vortexed and centrifuged (3,000 rpm for 3 min). The lower phase was transferred into a new Kimax® tube. The sample was evaporated under nitrogen flow which after it was dissolved in 300 μL of CHCl_3_.

Extracted fatty acids were fractionated by using a Bond Elut Silica cartridge. At first, the cartridge was activated by 6 mL of CHCl_3_-MeOH (1:1) mixture after which sample was added to the cartridge. The neutral lipid fraction was eluted with 8 mL of chloroform, and glycolipids were eluted with 8 mL of acetone, after which the fraction was discarded. Phospholipid fatty acids were eluted with 8 mL of methanol.

The PLFA fraction was evaporated to emptiness under nitrogen (N_2_) flow, which after it was dissolved to 1 mL of chloroform. 300 μL subsample was transferred into a pre-weighed tin cup and evaporated. The rest of the sample was stored at −20°C to wait for further processing. Chloroform was evaporated and the tin cup was weighted. δ^13^C of the PLFA sample was measured with a Thermo Finnigan DELTA^plus^Advantage *CF*-IRMS at the University of Jyväskylä, Finland. Based on quantified δ^13^C values of bulk PLFA sample, we calculated bacterial growth efficiency (BGE) as described in del Giorgio and Cole (1998) and the total decomposition rate for each substrate.

The rest of the divided PLFA fraction (700 μL) was evaporated under nitrogen flow after which 1 mL of hexane and 2 mL of 1% H_2_SO_4_ were added. Tubes were flushed under nitrogen flow for 5 s and incubated at 90°C for 90 min. After incubation, 1.5 mL of H_2_O and 4 mL of hexane were added. Tubes were vortexed and centrifuged at 3,000 rpm for 3 min. The upper phase was transferred into a new Kimax® tube. The collected phase was evaporated under nitrogen flow. The sample was dissolved by 500 μL of hexane and transferred into a small vial. The sample was still concentrated before analysis by evaporating it and dissolving it in 100 μL of hexane.

PLFAs were analyzed by combined gas chromatography and mass spectrometer (GC–MS). The length of a column (DB-23) was 30 metres and the diameter was 250 μM. The column film was 0.25 μM thick. The splitless mode was used for the mass spectrum. The injection temperature was 260°C. Total helium flow was 47.4 mL/min. The initial temperature of gas chromatography was 60°C and it was held for 1 min, after which the temperature was raised to 130°C and further to 180°C, and further to 220°C. The running time was 47 min per sample. Four different concentrations of the GLC Reference standard (Nu-Chek Prep Inc.) were prepared and analyzed to create a standard curve. Fatty acids were identified and integrated with GC Solution Postrun -software (Shimadzu). Based on the standard curve and recovery of internal standard, the amount of fatty acids in a sample was calculated as mg/g of carbon. After running PLFA samples on GC–MS, samples were evaporated under nitrogen flow and dissolved in 70 μL. Samples were sent to Austria where they were analyzed by using compound-specific isotope analysis (CSIA). The CSIA run was performed as in Taipale et al. (2019). Assimilation calculations are described in the Supplementary material.

### Decomposition rate and biochemical fate calculations

The decomposition rate of each substrate per year was calculated as a sum of the total mineralization rate per year and the assimilation rate per year. To convert this to months, the decomposition rate per year was divided by 12. Moreover, to determine total decomposition time as years or as months, 100 was divided by the percentual decomposition rate per year or month.

Proportional biochemical fate of decomposed substrate carbon in CO2, DIC, or biomass was calculated by dividing the mineralization or assimilation rates by total decomposition rate and multiplying it by 100 to get percent values.

### DNA and RNA analysis, and sequence data processing

At the end of the experiments, a 10–30 mL subsample of water was filtered through a pore size of 0.2 μM (Supor® 0.2 μM/25 mm, PES, Pall Corporation), and the filter was transferred into a bashing bead lysis tube (ZR BashingBead™ Lysis Tubes (0.1 & 0.5 mm), Nordic BioSite, United States) with 800 μL of DNA/RNA Shield™ (Zymo Research, United States). Samples were homogenized by bead-beating at 5.5 m/s for 40 s (Bead Ruptor Elite 24 bead mill homogenizer, Omni International Inc., United States).

DNA and RNA were simultaneously extracted by Chemagic™ 360 (PerkinElmer Inc., United States). For bacterial community analysis, cDNA was synthesized from RNA by using Maxima First-Strand Synthesis Kit with dsDNase (Thermo Fischer Scientific). Genomic DNA was eliminated by adding 0.5 μL of 10x dsDNase buffer and dsDNase and 1.5 μL of H_2_O and 2.5 μL of the sample. Samples were incubated at 37°C for 30 min after which it was incubated at 65°C for 5 min. During the last incubation, 0.5 μL of random hexamer primers and dNTPs and 1.5 μL of H_2_O were added. Samples were cool down to 4°C for 1 min after which 2 μL of 5x reverse transcriptase buffer and 0.5 μL of reverse transcriptase were added. Samples were incubated at 25°C for 10 min after which the temperature was raised to 50°C for 30 min, then heated to 85°C for 5 min and cooled down to 4°C.

PCR reactions were prepared by mixing 12.5 μL Maxima SYBRGreen/Fluorescein qPCR (2X) Master mix (Thermo Fisher Scientific, Lithuania. Polymerase: Hot Start Taq DNA polymerase), 2.5 μL of bovine serum album (BSA, 1 mg/mL), 1 μL of primers (10 μM P1-ITS4 and M13-ITS7 (Mäki et al., 2016) (Merck, CCTCTCTATGGGCAGTCGG-TGATCCTCCGCTTATTGATATGC and TGTAAAACGACGGCCAGTG-TGARTCATCGAATCTTTG, respectively) for fungi and P1-806R and M13-515FY (Merck, CCTCTCTATGGGCAGTCGG-TGATGGACTACNVGGGTWTCTAAT and TGTAAAACGACGGCCAGTG-TGYCAGCMGCCGCGGTAA, respectively) for bacteria) and 2 μL of extracted DNA or synthesized cDNA, which was used as a template for fungi and bacteria, respectively. PCR-grade H_2_O was added up to the total volume of 25 μL.

For the 16S rRNA gene, the PCR reaction was initialized with heating at 95°C for 3 min, followed by the denaturation phase at 95°C for 45 s, annealing at 50°C for 1 min, and elongation phase at 72°C for 90 s. Phases from denaturation to elongation were repeated 34 times after which the reaction was kept at 72°C for 10 min before cooling down to 4°C. For ITS, the PCR reaction was initialized with heating at 95°C for 3 min, followed by the denaturation phase at 95°C for 30 s, annealing at 55°C for 30 s, and the elongation phase at 72°C for 45 s. Phases from denaturation to elongation were repeated 35 times after which the reaction was kept at 72°C for 7 min before cooling down to 10°C. The size of the PCR amplicons was checked by running 5 μL of amplicons on 1% agarose gel (120 V for 45 min).

Sample barcoding was performed as PCR described above but using 1 μL of PCR amplicons from the first reaction as a template and with barcoded fusion primer IonA-M13F and P1-806R or IonA-M13F and P1-ITS4. The second PCR was started with heating samples up to 95°C for 3 min, followed by 10 cycles of denaturation at 95°C for 45 s, annealing at 54°C for 45 s and elongation at 72°C for 1 min. After 10 cycles, the temperature was kept at 72°C for 5 min before cooling down to 4°C. After the reaction, barcoded PCR amplicons were run on 1% agarose gel (120 V for 45 min). Barcoded PCR amplicon size was checked by agarose gel electrophoresis. Samples with low DNA content were purified and concentrated with SparQ Quanta purification beads (SparQ PureMag Beads, Quantabio) according to the manufacturer’s protocol. Amplicons were quantified with Qubit (Qubit Fluorometric Quantification, Thermo Fischer Scientific) and pooled equimolarly. Pooled library was purified by SparQ Quanta using 1.3x ratio (v/v), quantified by 2200 TapeStation system (Agilent Technologies, United States), and sequenced by Ion Torrent Personal Genome Machine (PGM) (Life Technologies, United States) using IonPGM Hi-Q View OT2 400 kit and Sequencing kit with a 318 IonChip (Thermo Fisher Scientific). Sequences were deposited to the NCBI Sequence Read Archive as project PRJNA810989.

Samples were sequenced by Ion Torrent Personal Genome Machine (PGM) (Life Technologies, United States) using IonPGM Hi-Q View OT2 400 kit and Sequencing kit with a 318 IonChip (Thermo Fisher Scientific). Sequences were deposited to the NCBI Sequence Read Archive as project PRJNA810989. More detailed DNA and RNA sample preparation are described in Supplementary material.

The sequence data from the IonTorrent server was processed by the CLC Microbial genomics module (CLC Genomic Workbench 12 with microbial genomics module, Qiagen, Denmark). 16S sequences were filtered based on the presence of both forward and reverse primers and ITS sequences based on the presence of the forward primer. In addition, sequences shorter than 250 bp and longer than 450 were discarded. The trimmed data was further subsampled using thresholds to 20000 and 18000 reads for 16S and ITS sequences, respectively. The minimum occurrence of 1 was set for OTUs before clustering and the creation of new OTUs with 80% taxonomic similarity was allowed. For the reference-based OTU clustering, the SILVA 16S v132 database at 99% resolution sequences and UNITE v7.2 database at 99% resolution were used for bacterial and fungal sequences, respectively. OTUs whose appearance was less than 1% were discarded from further data processing. “N/A” OTUs were checked to be fungi by aligning sequences against the RDP Classifier database (RDP Naive Bayesian rRNA Classifier Version 2.11, September 2015; UNITE fungal ITS trainset 07-04-2014) with an 80% confidence threshold.

### Statistical testing

PERMANOVA for microbial community and CSIA data was carried out in PRIMER7. Data were square root transformed and Bray–Curtis similarity was used to create resemblance tables. Non-metric multidimensional scaling (NMDS) analysis was performed to unravel microbial genera correlating with δ^13^C-values of PLFAs. Differences in mineralization rates, biomass assimilation, degradation rate, and water quality parameters were conducted by pair-wise PERMANOVA in PRIMER7. The used confidence level for all tests was 95%.

## Results

### Mineralization and assimilation rates of substrates and biochemical fate

**Figure 2:**
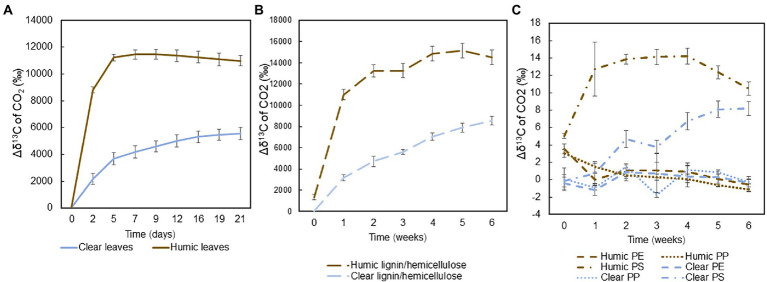
Averages and standard deviations of the mineralization of ^13^C-labeled **(A)** leaves (*n* = 4), **(B)** lignin-hemicellulose substrate (*n* = 4), and **(C)** microplastics during the experiments (*n* = 4) were calculated by subtracting average δ^13^C value of the control group from the δ^13^C of each treatment.

Mineralization of ^13^C-labeled substrates into the gas phase, indicative of microbial respiration, was measured during the experiments and calculated by subtracting the average δ^13^C value of the control group from the δ^13^C of each treatment: Mineralization_gas_ = δ^13^C_control_-δ^13^C_treatment_. Total ^13^C-labelled leaves and hemicellulose were rapidly mineralized in both humic and clear lake water, whereas recalcitrant lignin and microplastics were mineralized slowly or not at all. In humic lake water, mineralization of carbon, especially from leaves and hemicellulose, increased rapidly during the first week, after which the isotopic signal reached a steady-state (Figures 2A,B). In clear lake water, the mineralization of these natural substrates was not as rapid, and the ^13^C-enrichment increased during the whole experiment. Among plastic treatments, only the polystyrene carbon was mineralized during the experiment (Figure 2C).

**Figure 3:**
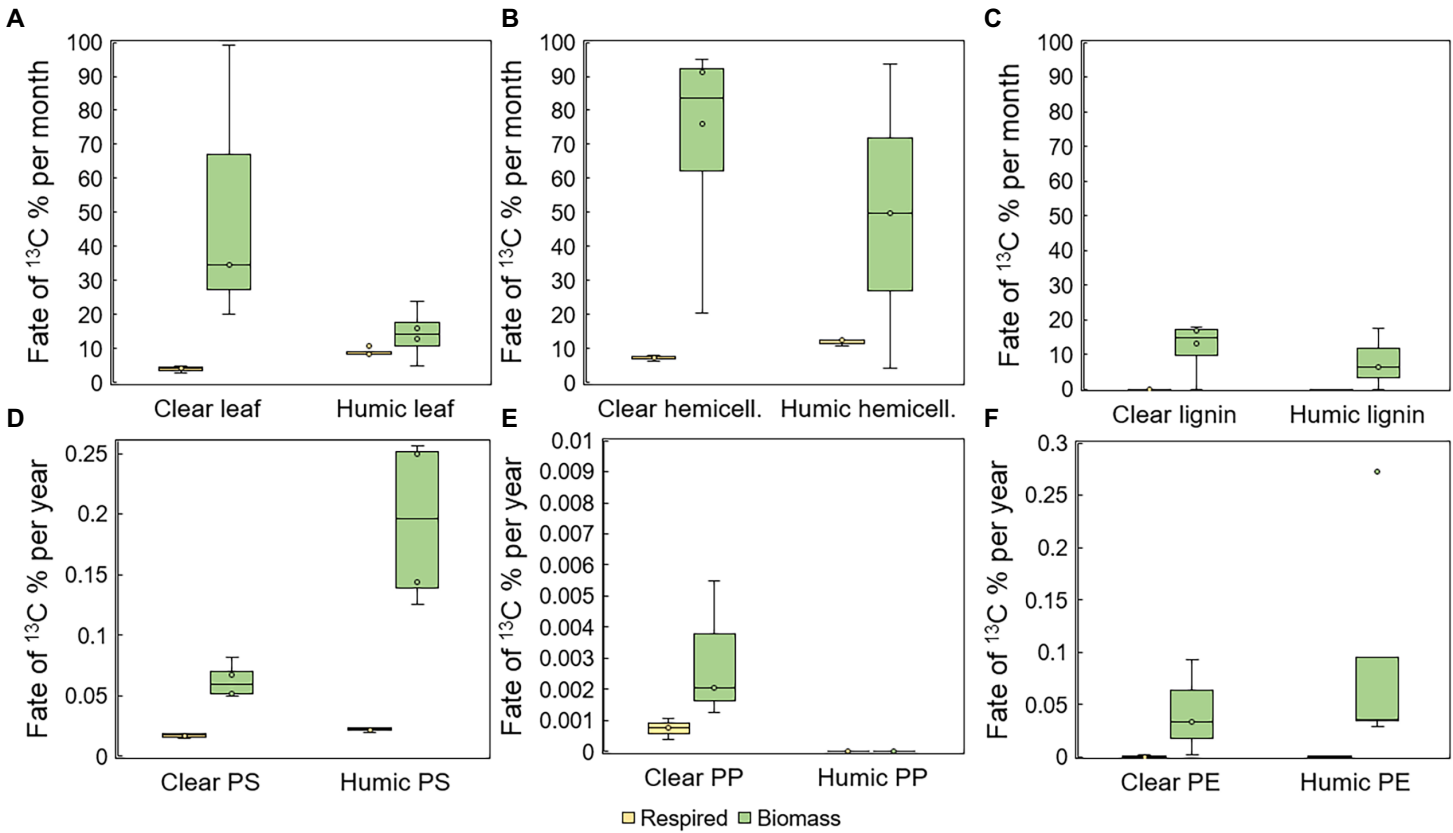
Assimilation and mineralization of ^13^C-labeled **(A)** leaves, **(B)** hemicellulose, **(C)** lignin, **(D)** PS, **(E)** PP, and **(F)** PE. In humic lake water *n* = 4 for leaf, PS, PP, and PE treatments and *n* = 3 for lignin and hemicellulose. In clear lake water *n* = 4 for lignin, hemicellulose, and PS treatments and *n* = 3 for leaf, PP, and PE.

Total mineralization rates were calculated at the end of the experiment. Leaves were mineralized at two times faster rate in humic (9.0 ± 1.2% per month) than in clear-lake water (4.0 ± 0.8% per month), and with statistically significant difference (pair-wise PERMANOVA: *p* = 0.028, *t* = 6.8505, Figure 3A). Hemicellulose was effectively mineralized during the experiment at the rate of 11.9 ± 0.9% and 7.3 ± 0.8% per month in humic and clear lake water, respectively (Figure 3B), whereas lignin from the added lignin-hemicellulose substrate was not mineralized at all (Figure 3C). Overall, microplastics were mineralized slowly or not at all (humic PS: 0.22 ± 0.02 ‰ per year; clear PS: 0.17 ± 0.02 ‰ per year; humic PP: not mineralized; clear PP 0.006 ± 0.004 ‰ per year; humic PE: 0.003 ± 0.006 ‰ per year; clear PE: 0.005 ± 0.009 ‰ per year; Figures 3D–F). Nevertheless, the mineralization rate of PS was significantly faster in humic lake water than in clear lake water (*p* = 0.026; *t* = 3.8699).

The assimilation rate of ^13^C-labeled substrate carbon into microbial biomass was quantified by measuring the ^13^C-enrichment and concentration of microbial PLFAs. Microbes utilized carbon from leaves at the rate of 14 ± 8% and 51 ± 42% per month in humic and clear lake water, respectively (Figure 3A). In addition, bacterial growth efficiency (BGE; (Steffens et al., 2015); Supplementary Figure S1) was significantly higher in clear than in humic lake water for leaves (*p* = 0.035, *t* = 2.920). Microbes utilized carbon from lignin at the rate of 8 ± 9% and 12 ± 8% per month in humic and clear lake waters, respectively. PS carbon was assimilated at the rate of 0.2 ± 0.1% and 0.1 ± 0.01% per year in humic and clear lake waters (Figure 3D) and the BGE of PS was significantly higher in humic than in clear lake water for (*p* = 0.032, *t* = 4.697). In humic lake water, PP carbon was not assimilated at all, whereas microbes assimilated PP carbon at the rate of 0.003 ± 0.002% per year in clear lake water. Microbes assimilated PE carbon at the rate of 0.1 ± 0.1% and 0.04 ± 0.05% per year in humic and clear lake waters, respectively (Figure 3F).

**Figure 4:**
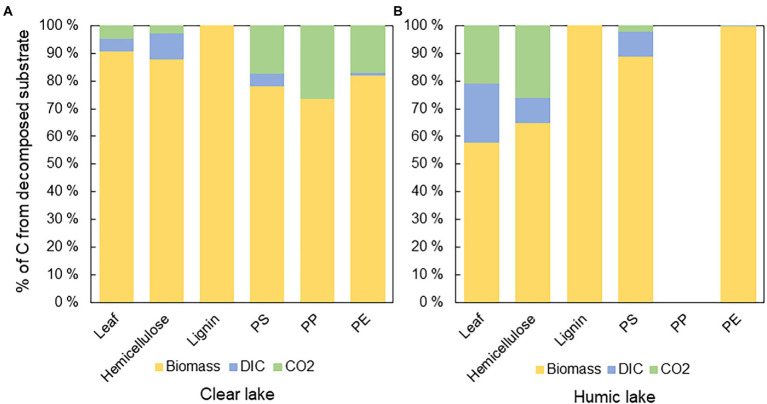
The fate of ^13^carbon from different substrates in **(A)** clear and **(B)** humic lake water. The major fate of ^13^C was in microbial biomass except in humic lake water with PP addition, where decomposition was not detected. In humic lake water *n* = 4 for leaf, PS, PP, and PE treatments and *n* = 3 for lignin and hemicellulose. In clear lake water *n* = 4 for lignin, hemicellulose, and PS treatments and *n* = 3 for leaf, PP, and PE.

The biochemical fate of substrate-derived carbon was calculated by combining stable isotope measurements of microbial biomass, CO_2_, and DIC, and calculating percentual proportions of decomposed substrate carbon in each end-product. In all treatments, substrate carbon was assimilated into microbial biomass rather than respired. However, in humic lake water, only 58 ± 14% of decomposed ^13^C-leaves, and 65 ± 33% of ^13^C-hemicellulose were assimilated into microbial biomass, whereas in clear lake water, 91 ± 4% of decomposed ^13^C-leaves and 88 ± 8% of ^13^C-hemicellulose were assimilated into biomass (Figure 4A). In contrast, all the decomposed lignin carbon ended up in microbial biomass in both lake waters. ^13^C-microplastics were also mainly assimilated into biomass (PS humic 89 ± 4%; PS clear 78 ± 3%; PP humic not decomposed; PP clear 74 ± 17%; PE humic 99.9 ± 0.2%; PE clear 82 ± 31%; Figure 4B).

Decomposition rates of natural substrates did not differ between studied lake waters, whereas PS and PE were decomposed faster in humic lake water (Table 1). Leaves were decomposed within 5 ± 2 months in humic and 3 ± 2 months in clear lake water. Hemicellulose was decomposed at the same speed as leaves from 1 to 6 months and from 1 to 2 months in humic and clear lake water, respectively, whereas lignin from hemicellulose was decomposed within 1 or 2 years or was not decomposed at all during the experiment. PS was decomposed within 500 ± 150 years in humic and 1,300 ± 250 years in clear lake water. PP was not decomposed at all in humic lake water and in clear lake water its decomposition rate was defined to be higher than 10,000 years. In contrast, PE was decomposed within 2,350 ± 1,350 years in humic lake water whereas in clear lake water PE decomposition rate is at least 1,100 years but varies up to over 10,000 years, similarly to PP decomposition.

**Table 1:** Decomposition rates and main decomposers of different substrates in humic and clear lake water.

Substrate	Decomposition rate as years or months (humic)	Decomposition rate as years or months (clear)	Main decomposers
Leaf	5 ± 2 months	3 ± 2 months	Bacteria (Burkholderiaceae and *Arcicella* sp.)
Lignin-hemicellulose	See separately below	See separately below	Bacteria
Hemicellulose	From 1 to 6 months	From 1 to 2 months	Not separated from lignin-hemicellulose
Lignin	1 year or not degraded at all	2 ± 1 or not degraded at all	Not separated from lignin-hemicellulose
PS	500 ± 150 years	1,300 ± 250 years	Alpha- and Gammaproteobacteria; potentially family Burkholderiaceae
PP	Not degraded	>10,000 years	Planctomycetes
PE	2,350 ± 1,350 years	From 1,100 to >10,000 years	Planctomycetes

### Identification of substrate decomposers by combining community data and CSIA

**Figure 5:**
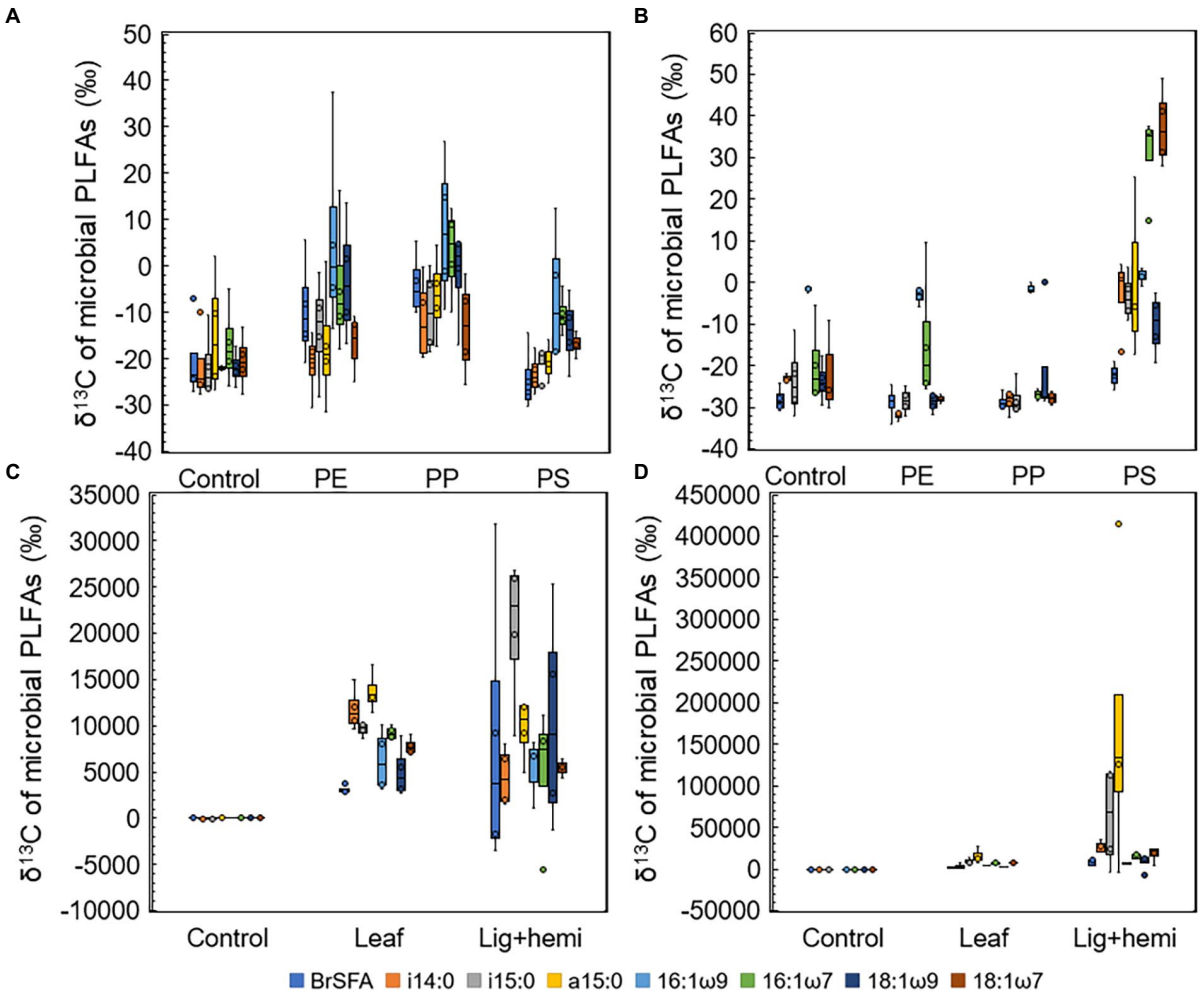
δ^13^C-values of microbial PLFAs in **(A)** humic lake water microplastic treatments, **(B)** clear lake water microplastic treatments, **(C)** humic lake water natural substrate treatments, and **(D)** clear lake water natural substrate treatments.

**Figure 6:**
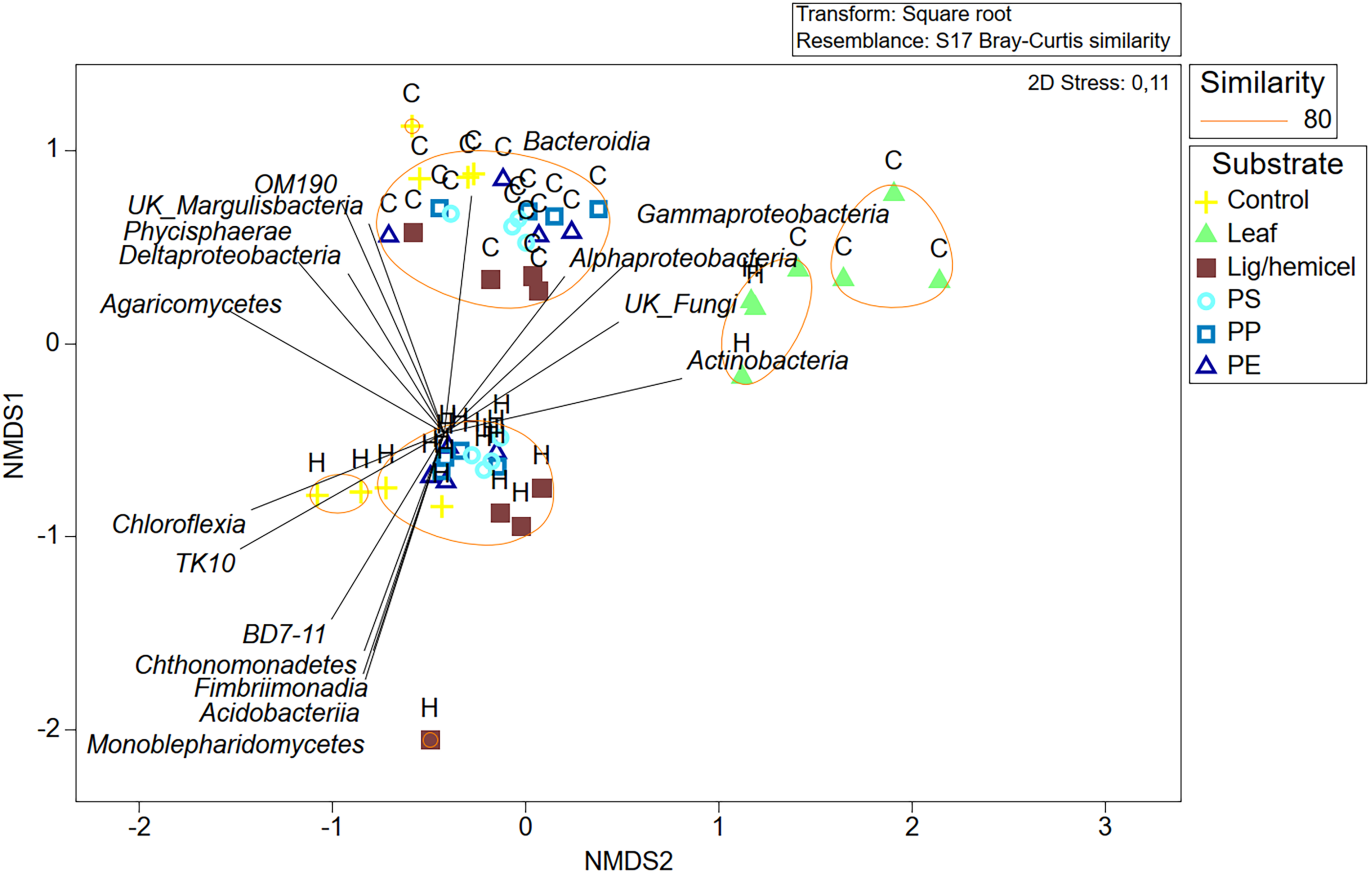
Non-metric multidimensional scaling plot of Bray–Curtis similarity of standardized and square root transformed OTU data at the class level. Letter H indicates humic lake water and letter C indicates clear lake water.

Differences between δ^13^C-values of treatments and controls were used to identify the most efficient decomposers of each substrate (Figures 5A–D). In addition, to identify decomposers more accurately, assimilated ^13^C from the added substrate to each PLFAs was calculated as Δδ^13^C = δ^13^C-PLFA_treatment_ - δ^13^C-PLFA_control_ and Δδ^13^C-results were combined with microbial community data in NMDS ordination analysis (Figure 6). In clear lake water leaf treatment, BrSFAs, i15:0, a15:0, 16:1ω7, 18:1ω9, and 18:1ω7 had higher δ13C-values in comparison to control (*p* = 0.033, *t =* 2.22; *p* = 0.031, *t* = 5.54; *p* = 0.034, *t* = 5.59; *p* = 0.026, *t* = 10.86; *p* = 0.034, *t* = 15,48; *p* = 0.027, *t* = 4.63). In humic lake water leaf treatment, all detected PLFAs had higher δ^13^C-values than the control (*p* < 0.05). NMDS ordination analysis of leaf samples showed that especially Δδ^13^C values of 16:1ω7, i14:0, 18:1ω9, BrSFA, 16:1ω9, and 18:1ω7 correlated (Pearson correlation) with humic lake water samples. Several bacterial genera correlated with humic lake water samples and were identified to belong to Alpha-, Delta-, and Gammaproteobacteria, Bacteroidetes, Planctomycetes, Verrucomicrobia, and Chloroflexi. Δδ^13^C values of 16:1ω7, which is characteristic for Alpha- and Gammaproteobacteria and major fatty acid of *Arcicella* sp. (Bacteroidetes) (Kämpfer et al., 2009; Hahn et al., 2010; Taipale et al., 2019), correlated with five genera from the family Burkholderiaceae, and *Arcicella* sp. (Bacteroidetes). In addition, the involvement of two uncultured Planctomycetes in the decomposition process of leaves in humic lake water was exposed by the correlation of high Δδ^13^C values of 18:1ω9 with Planctomycetes OTUs. Only one fungal genus *Rhodotorula* sp. (Basidiomycota) correlated with Δδ^13^C values of 18:1ω9, characteristic to fungi Planctomycetes (Willers et al., 2015; Taipale et al., 2019).

In clear lake water lignin-hemicellulose treatment PLFAs i14:0, a15:0, 16:1ω7, and 18:1ω7 had higher δ^13^C-values than control (*p* = 0.041, *t* = 3.26; *p* = 0.041, *t* = 2.01; *p* = 0.026, *t* = 3.32; *p* = 0.029, *t* = 4.00). In contrast, only δ^13^C-values of i14:0, i15:0, and a15:0 differed from control in humic lake water lignin-hemicellulose treatment (*p* = 0.025, *t* = 2.74; *p* = 0.034, *t* = 4.96; *p* = 0.023, *t* = 5.64). Only bacterial taxa correlated towards high Δδ^13^C values in lignin-hemicellulose treatment. These bacterial genera belonged to Bacteroidetes, Planctomycetes, Alphaproteobacteria, and Verrucomicrobia.

In humic PS treatment, 16:1ω9 was the only PLFA whose δ^13^C-values differed from the control (*p* = 0.031, *t* = 2.68). In contrast, δ^13^C-values of i14:0, i15:0, 16:1ω9, 16:1ω7, 18:1ω9, and 18:1ω7 in clear lake water PS treatment differed from control (*p* = 00.27, *t* = 4.27; *p* = 0.019, *t* = 3.77; *p* = 0.047, *t* = 3.05; *p* = 0.025, *t* = 6.95; *p* = 0.043, *t* = t = 3.07; *p* = 0.031, *t* = 7.89, respectively). The highest δ^13^C-values were observed in 16:1ω7 and 18:1ω7, characteristic to Alpha- and Gammaproteobacteria (Taipale et al., 2019). NMDS ordination analysis for lake waters with PS addition showed that high Δδ^13^C values of 16:1ω7 and 18:1ω7 correlated highly (>0.9) with seven genera from class Alphaproteobacteria, identified as *Elstera* sp., *Novosphingobium* sp., AT-s3-44 (Sneathiellaceae), uncultured Rhodospirillales, *Hirschia* sp., *Reyranella* sp., and *Bosea* sp., and six genera belonging to Gammaproteobacteria, identified as *Malikia* sp., *Pelomonas* sp., *Hydrogenophaga* sp., *Hydrocarboniphaga* sp., uncultured Burkholderiaceae, and *Polynucleobacter* sp. Among Gammaproteobacteria, five of six potential PS decomposers belong to the family Burkholderiaceae. Moreover, highly positive regression (R^2^ = 0.986) was exposed between the amount of detected *Hydrocarboniphaga* sp. (Gammaproteobacteria) sequences and mineralization rate in clear lake water (Supplementary Figure S3).

In clear PE and PP treatments, only i14:0 had significantly higher δ^13^C-values in comparison to control (*p* = 0.026, *t* = 5.38; *p* = 0.027, *t* = 4.03, respectively). In humic PE and PP treatments, 16:1ω9 and 18:1ω9 had higher δ^13^C-values in comparison to control (PE: *p* = 0.026, *t* = 3.01; *p* = 0.03, *t* = 2.74; PP: *p* = 0.032, *t* = 4.04; *p* = 0.03, *t* = 3.65, respectively) 16:1ω9 is typical to Spumella-like flagellates (Taipale et al., 2019) and 18:1ω9 is characteristic to Planctomycetes and fungi (Willers et al., 2015; Taipale et al., 2019), suggesting that these microbial groups can decompose aliphatic microplastics in humic lakes. NMDS ordination analysis showed that these PLFAs correlated with two and three fungal OTUs from phylum Basidiomycota in PP and PE treatments, respectively. Furthermore, high Δδ^13^C values of 18:1ω9 correlated with *Singulisphaera* sp., I-8 (Phycisphaeraceae), and an uncultured BD7-11 in the PP treatment and with uncultured Pirellulaceae, *Singulisphaera* sp., I-8 (Phycisphaeraceae), and uncultured BD7-11 in PE treatment.

## Discussion

### Mineralization and assimilation rates are dependent on substrate recalcitrance

Leaves, hemicellulose, and PS were mineralized faster in humic than in clear lake water, whereas other substrates were mineralized in both studied lake waters with similar rates. Environmental parameters shape aquatic microbial community structure and affect microbial ability to utilize different carbon sources (Wang et al., 2018; Yang et al., 2020; Meng et al., 2022). For example in humic lakes, heterotrophic microbial lifestyle dominates over autotrophy (Jansson et al., 2007), which likely increases competition over carbon sources and favors microbes that can effectively utilize carbon sources when they enter a lake ecosystem. Thus, humic lake water microbes have adapted to utilize easy carbon sources fast after they enter lake water. In contrast, clear lakes have naturally more labile organic carbon sources (Taipale and Sonninen, 2009), which could explain why the mineralization rates of the added easily degradable substrates were slower in clear lake water. Nevertheless, in both studied lake waters, high mineralization rates of leaves and hemicellulose indicated that easily degradable carbon sources are rapidly utilized by freshwater microbes and respired. In addition, also PS carbon can be mineralized although the rate is very low.

Polymers containing aromatic rings are highly recalcitrant and common in humic substances and thus commonly present in humic aquatic ecosystems. Since humic lakes are typically poor in labile carbon sources (Hessen and Tranvik, 1998), we hypothesized that humic lake water microbes have adapted to utilize recalcitrant aromatic compounds. Against our hypothesis, decomposition rates of PE and PS did not differ significantly in clear lake water. However, the decomposition rate of aromatic PS was faster than the decomposition of aliphatic PE and PP in humic lake water. Unlike PE and PP, PS is denser than water, which allows it to submerge (Anderson et al., 2016) and microbes to attach to its surface more easily. In nature, polystyrene can end up in sediment (Anderson et al., 2016), where also anaerobic microbes could utilize it as a carbon source. Decomposition rates of microplastic carbon in nature can be higher than what our results suggest since photodegradation increases plastic degradation in water (Tian et al., 2019) together with thermo-oxidation (Anderson et al., 2016).

Our results show that natural carbon substrates – even lignin – are utilized faster than synthetic polymer carbon in both lake water types. The mineralization rates of studied substrates indicated that humic lake water microbes can be more efficient to mineralize both easier and more recalcitrant carbon sources than clear lake water microbes are. However, only PS carbon was assimilated faster into microbial biomass in humic than in clear lake water, whereas other substrates were equally assimilated into microbial PLFAs in clear and humic lake waters. In addition, the assimilation rate of PS carbon was not significantly different in comparison to PE. It seems that PS is converted more efficiently to CO_2_ than PE, whereas PS and PE carbon are preferably and equally well utilized in a structural cell component. However, in this study, we measured only ^13^C assimilation into PLFAs. Since some amino acids, e.g., phenylalanine and tyrosine, contain aromatic rings, one could assume that polymers containing aromatic rings are processed rather *via* the amino acid cycle due to the similar structures of these compounds. Thus, other assimilation pathways such as the amino acid cycle should be considered in future research as well.

### The major biochemical fate of decomposed substrate carbon is microbial biomass

The chemical structure and recalcitrance of a carbon substrate play an important role in decomposition and affect its biochemical fate in nature. In accordance with our hypothesis, the major fate of all studied substrates’ carbon that was decomposed was in microbial biomass, whereas mineralization was found to play only a minor role in the decomposition process of microplastics and lignin. Similar results were found for PE decomposition by the freshwater microbial community (Taipale et al., 2022) but not for PS decomposition by mealworms which used only minor amounts for biomass formation (Yang et al., 2015). Although the mineralization of substrate carbon covers only a small proportion of all carbon fate, measurement of δ^13^C-values from gas could be used as an indicator for decomposition, since it is relatively easy, fast, and cheap to measure in comparison to CSIA. Nevertheless, the measurement of both mineralization and assimilation is necessary when the aim is to quantify decomposition rates.

### Microbes behind the decomposition process

Bacteria are known to dominate the early-stage decomposition of plant litter due to their faster growth rates and better competition compared to fungi (Ágoston-Szabó et al., 2006) Similarly, our results showed that bacteria were the main decomposers of leaves at the early-stage decomposition process. Several PLFAs had high δ^13^C values, but especially *Arcicella* sp. (Bacteroidetes) and five genera from the family Burkholderiaceae were identified as the major decomposer of leaves (Table 1). Moreover, bacteria are more efficient to decompose cellulose and lignin-hemicellulose than fungi in semiarid soils (Torres et al., 2014). Our results suggested that carbon from lignin-hemicellulose was assimilated mainly by bacteria also in aquatic systems. However, it should be noted that since hemicellulose is a more easily degradable substrate than lignin, it is likely that the δ^13^C signal originated mainly from hemicellulose and only a small proportion originated from lignin, whose carbon was also shown to be assimilated into microbial biomass. Therefore, the importance of fungi in the decomposition of lignin itself cannot be excluded.

In clear lake water PS treatment, δ^13^C values of several PLFAs differed from control and therefore indicated that several microbial groups are capable of decomposing PS. Our results suggested that Gammaproteobacteria, especially the family Burkholderiaceae, play potentially an important role in the decomposition process of polystyrene. Burkholderiaceae are known to be able to degrade aromatic compounds (Pérez-Pantoja et al., 2012) and a member of Burkholderiaceae, *Ideonella sakaiensis*, has been found to decompose polyethylene terephthalate (PET), which contains aromatic and heteroatomic structures (Yoshida et al., 2016). In humic lake water, 16:1ω9 was the only fatty acid whose δ^13^C values were significantly different in comparison to control in humic lake water. 16:1ω9 is characteristic to eukaryotic Spumella-like flagellates, which were found to participate in PE decomposition in humic lake water (Taipale et al., 2019). Thus, our results suggest that Spumella-like flagellates could participate also in the decomposition process of PS, although it cannot be confirmed since other Eukaryota except fungi were not studied. Despite of that fungi have been recognized as potential plastic decomposers (Sánchez, 2020), fungal participation in the decomposition of PS particles was not observed in our study.

In humic lake water PE and PP treatments, δ^13^C values of PLFAs 16:1ω9 which is characteristic to Spumella-like flagellates, and 18:1ω9, typical to fungi and Planctomycetes, differed from control. The absence of fungal biomarker 18:2ω6 suggests that Planctomycetes have a more likely ability to assimilate PE and PP carbon in comparison to fungi. Thus, against our hypothesis, fungi were not contributing to the decomposition of microplastics. However, decomposition rates of PP and PE were extremely slow in contrast to earlier studies (Taipale et al., 2019, 2022), and the assimilation of PP and PE carbon into PLFAs was likely inhibited by the presence of other easier carbon sources in lake water. Therefore, it is plausible that several freshwater bacteria can assimilate aliphatic microplastic carbon, but the high recalcitrance of microplastics and the access to easier natural carbon sources make it an unfavorable carbon source and it is therefore slowly decomposed.

## Data availability statement

The datasets presented in this study can be found in online repositories. The names of the repository/repositories and accession number(s) can be found at: https://www.ncbi.nlm.nih.gov/, SRX14310857- SRX14310911.

## Author contributions

ST designed the research. JV performed the research and wrote the manuscript. RN helped with bioinformatics. MT contributed microbial community analyses. MK and MP contributed and performed the CSIA. All authors discussed the results and commented on the manuscript.

## Funding

This research was funded by the Kone Foundation grant 201905367 awarded to the ST, Academy of Finland grant 333564 awarded to ST, and Academy of Finland 325107 awarded to MT.

## Conflict of interest

The authors declare that the research was conducted in the absence of any commercial or financial relationships that could be construed as a potential conflict of interest.

## Publisher’s note

All claims expressed in this article are solely those of the authors and do not necessarily represent those of their affiliated organizations, or those of the publisher, the editors and the reviewers. Any product that may be evaluated in this article, or claim that may be made by its manufacturer, is not guaranteed or endorsed by the publisher.
