# Supplementary material for: Decomposition rate and biochemical fate of carbon from natural polymers and microplastics in boreal lakes
Source: Front Microbiol. 2022 Nov 8;13:1041242. doi: 10.3389/fmicb.2022.1041242 (PMC9679218; doi:10.3389/fmicb.2022.1041242)
Supplement: Supplementary file 1 [file Data_Sheet_2.PDF]

Supplementary Material

1. Supplementary Methods

1. Mineralization rate calculations

The concentration of carbon as mg/l was calculated as follows:

c_C=((TCD_{CO2}*($V_{sample+helium}$/V_{sample})*M_C)/1000/M_{CO2})/(273.15/$T_{experiment}$),

where TCD_{CO2}=TCD detector measurement results for the amount of CO_2 in the tube as ppm, $V_{sample+Helium}$=summed volume of helium and sample gas in the Exetainer® tube, V_{sample}=volume of the gas sample, M_C=molar mass of carbon, M_{CO2}=molar mass of carbon dioxide and $T_{experiment}$=temperature of the experiment as kelvins. The mass of carbon in the gas and the water phase of the bottle was further calculated:

m_C=c_C*V_{phase},

where V_{phase}=volume of the water or gas phase in the bottle. $\delta^{13}C$ values of CO_2 and DIC were analyzed using an Isoprime TraceGas pre-concentrator unit connected to an Isoprime IRMS (Isoprime100 IRMS, Elementar UK Ltd., Cheadle, UK) at the University of Jyväskylä, Finland. $\delta^{13}C$ values were drift corrected and two-point calibrated based on external standards. $\delta^{13}C$ values were turned to atom% by using the formula (1):

AP=100*($\delta^{13}C$+1000)/[$\delta^{13}C$+1000+(1000/$R_{standard}$)],

where $R_{standard}$ value is 0.01118 (VPDB). To calculate the difference between treatments and control, the average of $AP_{Control}$ was subtracted from the AP of each sample:

δAP $Miner\%_{experiment}$ =$AP_{Treatment}$- $AP_{Control\ average}$

Thus, positive differences indicated that ^{13}C was released from the added ^{13}C-substrate whereas negative differences indicated that the substrate was not degraded. Percentual mineralization of ^{13}C-substrate in gas or water phases during the experiment was calculated by a formula:

=((δAP/100)*m_{CO2})/($m_{added\ substate}$*$C\%_{added\ substrate}$*$^{13}C\%_{added\ substrate}$)*100,

where m_{CO2}=amount of CO_2 in the gas or the amount of DIC in the water as mg, $m_{added\ substate}$=weight of the added substrate as mg, $C\%_{added\ substrate}$=proportion of carbon molecular weight from the total molecular weight of the added substrate, and $^{13}C\%_{added\ substrate}$=enrichment percentage of labeled substrate's carbon. To calculate the mineralization of ^{13}C-substrate during a year, we then calculated:

$Miner\%_{year}$=$Miner\%_{experiment}$/t_{days}*365.25,

where t_{days}=duration of the experiment as days.

Lignin involves approximately 20 % of impurities which are mostly carbohydrates, including hemicellulose (3). Since hemicellulose is an easier carbon source it is probably consumed before microbes start to utilize lignin as a sole carbon source. Thus, we needed to extract the $\delta^{13}C$-value

originating from hemicellulose degradation from the degradation of lignin-hemicellulose. Amount of added ^{13}C-hemicellulose and ^{13}C-lignin as mg was calculated:

$m_{13C\ hemicellulose}=m_{(added\ 13C)}*0.2$ and $m_{13Clignin}=m_{(added\ 13C)}*0.8$

where $m_{(added\ 13C)}$=mass of added lignin-hemicellulose multiplied with carbon content and proportion of labeled carbon of the total carbon in the substrate.

2. Assimilation rate calculations

The coefficient to evaluate the proportion of PLFA from total biomass was calculated for each sample:

$coeff_{biomass}=(m_{empty}-m_{sample})*(1/0.3)/m_{filter}$,

where m_{empty}=mass of the empty tin cup as mg, m_{sample}=mass of the tin cup after adding and evaporating PLFA fraction as mg, and m_{filter} is the mass of FA sample filter after freeze-drying as mg.

To estimate microbial biomass as a carbon in each bottle, we calculated:

$m_{biomass}$ (mg)= $((m_{empty}-m_{sample})*V_{water\ phase}/V_{filtrated})*coeff_{biomass}*0.5$,

where $V_{water\ phase}$=volume of water in the bottle, and $V_{filtrated}$=volume of filtrated water. The coefficient 0.5 was used since approximately 50 % of microbial biomass is carbon (2).

Similarly than with mineralization calculations, the percentual assimilation of ^{13}C-substrate into biomass during the experiment was calculated by a formula:

$Assim\%_{experiment}=((\delta AP/100)*m_{biomass})/(m_{added\ substate}*C\%_{added\ substrate}*^{13}C\%_{added\ substrate})*100$.

and

$Assim\%_{year}=Assim\%_{experiment}/t_{days}*365.25$

to calculate the assimilation of ^{13}C-substrate carbon into biomass per year.

2. Supplementary Figures and Tables

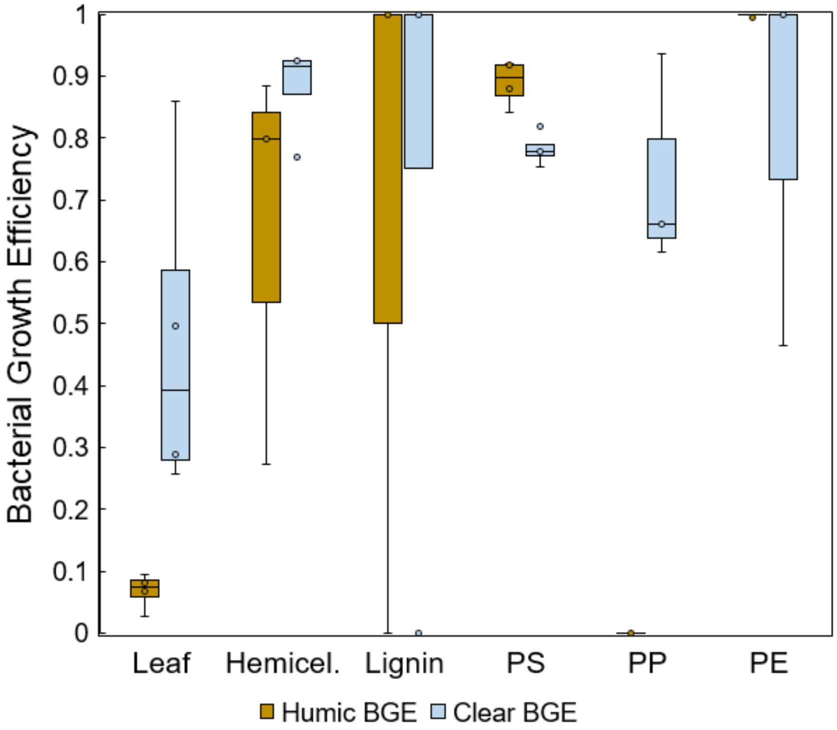

Supplementary figure 1. Bacterial growth efficiency (BGE) in humic and clear lake waters. Humic leaves n=4, clear n=3; humic lignin n=3 and clear n=4; a microplastics in humic n=4 and clear lake waters PS n=4, PP and PE n=3.

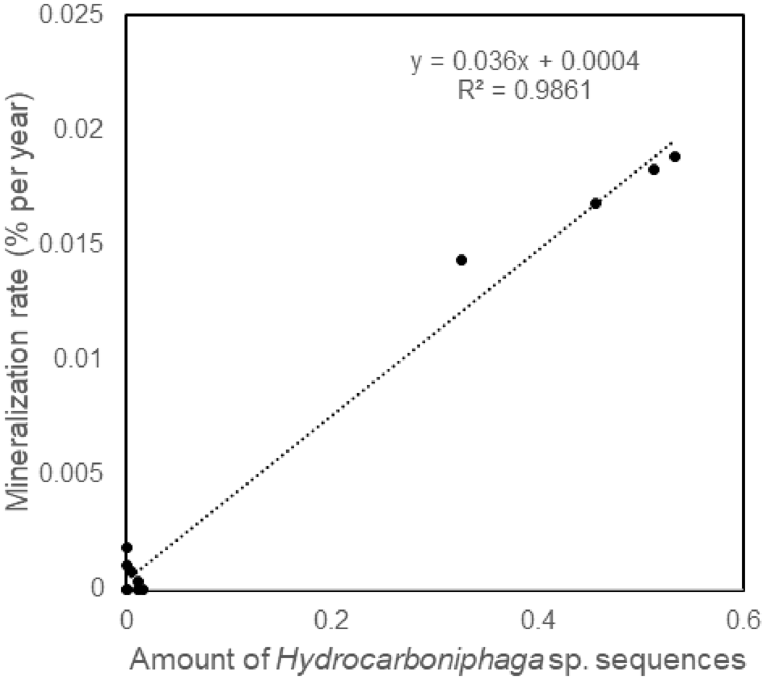

Supplementary figure 2. Linear regression analysis between mineralization rates of ^{13}C-plastics and the relative abundance of *Hydrocarboniphaga* sp. sequences in clear lake water.

Table S1. Pearson correlations of OTU results at class, family, and genus levels, assimilation of ^{13}C-leaves to phospholipid fatty acids, with NMDS1 and NMDS2 axes.

**CLASS**	**FAMILY**	**GENUS**	**NMDS1**	**NMDS2**
Gammaproteobacteria	Burkholderiaceae	UK_Burkholderiaceae	-0.9755	0.03307
16:1ω7	16:1ω7	16:1ω7	-0.9715	0.21905
Gammaproteobacteria	Burkholderiaceae	Polynucleobacter	-0.9709	0.06678
Bacteroidia	Spirosomaceae	Arcicella	-0.9704	0.06727
i14:0	i14:0	i14:0	-0.9634	-0.1455
Gammaproteobacteria	Burkholderiaceae	Leptothrix	-0.956	0.00217
Alphaproteobacteria	Acetobacteraceae	Rhodovastum	-0.9381	0.10277
Alphaproteobacteria	Rhodobacteraceae	Rhodobacter	-0.9248	0.06972
Deltaproteobacteria	0319-6G20	UK_0319-6G20	-0.9175	0.09069
Gammaproteobacteria	Burkholderiaceae	Limnohabitans	-0.9139	0.30328
Gammaproteobacteria	Burkholderiaceae	Sphaerotilus	-0.9134	0.04927
Bacteroidia	37-13	UK_37-13	-0.8839	0.03734
18:1ω9	18:1ω9	18:1ω9	-0.835	0.05732
vadinHA49	UK_vadinHA49	UK_vadinHA49	-0.8348	-0.0322
Verrucomicrobiae	Pedosphaeraceae	Pedosphaera	-0.8292	0.0604
Alphaproteobacteria	Acetobacteraceae	Acidisoma	-0.8062	-0.0127
Bacteroidia	Chitinophagaceae	Heliimonas	-0.7866	-0.0155
Verrucomicrobiae	Pedosphaeraceae	UK_Pedosphaeraceae	-0.7773	0.02188
Acidobacteriia	Solibacteraceae (Subgroup 3)	Candidatus Solibacter	-0.7745	0.04313
Deltaproteobacteria	Archangiaceae	Anaeromyxobacter	-0.7402	-0.0764
Gammaproteobacteria	Cellvibrionaceae	Cellvibrio	-0.7289	-0.0111
Verrucomicrobiae	Verrucomicrobiaceae	Prosthecobacter	-0.7212	-0.4551
BrSFA	BrSFA	BrSFA	-0.6945	0.67305
Phycisphaerae	Phycisphaeraceae	UK_Phycisphaeraceae	-0.6911	0.03763
16:1ω9	16:1ω9	16:1ω9	-0.6852	0.49028
Chloroflexia	Roseiflexaceae	UK_Roseiflexaceae	-0.6468	-0.1099
Microbotryomycetes	Sporidiobolaceae	Rhodotorula	-0.6369	-0.093
18:1ω7	18:1ω7	18:1ω7	-0.6014	0.41585

Table S2. Pearson correlations of OTU results at class, family, and genus levels, assimilation of ^{13}C-lignin-hemicellulose to phospholipid fatty acids, with NMDS1 and NMDS2 axes.

**CLASS**	**FAMILY**	**GENUS**	**NMDS1**	**NMDS2**
i14:0	i14:0	i14:0	0.90824	0.26964
a15:0	a15:0	a15:0	0.90425	0.12694
16:1ω7	16:1ω7	16:1ω7	0.89304	0.24392
18:1ω7	18:1ω7	18:1ω7	0.88831	0.12568
i15:0	i15:0	i15:0	0.8735	-0.0449
18:1ω9	18:1ω9	18:1ω9	0.79979	-0.4832
BrSFA	BrSFA	BrSFA	0.74565	-0.4346
Bacteroidia	UK_Chitinophagales	UK_Chitinophagales	0.64591	0.6871
Phycisphaerae	Phycisphaeraceae	UK_Phycisphaeraceae	0.62659	0.44955
Alphaproteobacteria	Rhodobacteraceae	Tabrizicola	0.61989	0.66238
Bacteroidia	Spirosomaceae	Emticicia	0.60465	0.70457
UK_Margulisbacteria	UK_Margulisbacteria	UK_Margulisbacteria	0.60405	0.72638
Verrucomicrobiae	Verrucomicrobiaceae	Prosthecobacter	0.60133	0.62189

Table S3. Pearson correlations of OTU results at class, family, and genus levels, assimilation of ^{13}C-polystyrene to phospholipid fatty acids, with NMDS1 and NMDS2 axes.

CLASS	FAMILY	GENUS	NMDS1	NMDS2
Verrucomicrobiae	Verrucomicrobiaceae	Prosthecobacter	0.99377	-0.0619
Gammaproteobacteria	Burkholderiaceae	Malikia	0.98872	-0.0025
Alphaproteobacteria	Elsteraceae	Elstera	0.98737	-0.0742
Gammaproteobacteria	Burkholderiaceae	Pelomonas	0.98716	-0.1016
Planctomycetacia	Gemmataceae	UK_Gemmataceae	0.98715	-0.0868
Gammaproteobacteria	Burkholderiaceae	Hydrogenophaga	0.98476	-0.0386
18:1ω7	18:1ω7	18:1ω7	0.98361	-0.0447
Alphaproteobacteria	Sphingomonadaceae	Novosphingobium	0.98275	0.06472
Bacteroidia	Saprospiraceae	UK_Saprospiraceae	0.98004	0.0154
OM190	UK_OM190	UK_OM190	0.97994	0.02698
Alphaproteobacteria	Sneathiellaceae	AT-s3-44	0.97934	0.01064
Planctomycetacia	Rubinisphaeraceae	UK_Rubinisphaeraceae	0.97895	-0.0152
Gammaproteobacteria	Solimonadaceae	Hydrocarboniphaga	0.97525	-0.0066
Planctomycetacia	Gemmataceae	Zavarzinella	0.9751	0.02621
Deltaproteobacteria	UK_Bradymonadales	UK_Bradymonadales	0.97426	-0.1441
Alphaproteobacteria	UK_Rhodospirillales	UK_Rhodospirillales	0.97318	0.04561
Bacteroidia	Spirosomaceae	Emticicia	0.97176	0.03087
Verrucomicrobiae	Verrucomicrobiaceae	UK_Verrucomicrobiaceae	0.97169	0.02978
Acidobacteriia	Solibacteraceae (Subgroup 3)	Paludibaculum	0.97164	-0.0015
Deltaproteobacteria	UK_PB19	UK_PB19	0.96887	0.04332
Deltaproteobacteria	mle1-27	UK_mle1-27	0.96636	0.08095
Planctomycetacia	UK_Planctomycetales	UK_Planctomycetales	0.96382	0.11847
16:1ω7	16:1ω7	16:1ω7	0.95874	0.18894
Bacteroidia	37-13	UK_37-13	0.9525	0.08308
Actinobacteria	Microbacteriaceae	Herbiconiux	0.94424	0.04128
Phycisphaerae	Phycisphaeraceae	SM1A02	0.94327	0.09091
Alphaproteobacteria	Hyphomonadaceae	Hirschia	0.9404	0.05034
Gammaproteobacteria	Burkholderiaceae	UK_Burkholderiaceae	0.93928	-0.0083
Gammaproteobacteria	Burkholderiaceae	Polynucleobacter	0.92993	-0.0739
Alphaproteobacteria	Reyranellaceae	Reyranella	0.92949	-0.0525
i15:0	i15:0	i15:0	0.92625	0.15735
Phycisphaerae	Phycisphaeraceae	CL500-3	0.91893	0.17459
UK_Margulisbacteria	UK_Margulisbacteria	UK_Margulisbacteria	0.91449	0.12876
Alphaproteobacteria	Beijerinckiaceae	Bosea	0.90903	-0.157
Deltaproteobacteria	Polyangiaceae	Pajaroellobacter	0.90503	0.1576
Gammaproteobacteria	Burkholderiaceae	Limnobacter	0.89808	0.10385
Phycisphaerae	Phycisphaeraceae	UK_Phycisphaeraceae	0.89663	-0.1309
Alphaproteobacteria	Rhodobacteraceae	Tabrizicola	0.88184	-0.0953
i14:0	i14:0	i14:0	0.87709	-0.3149
Gammaproteobacteria	Burkholderiaceae	Curvibacter	0.86249	0.33631

Chlamydiae	Simkaniaceae	UK_Simkaniaceae	0.8507	0.11381
Alphaproteobacteria	Azospirillaceae	Azospirillum	0.82615	-0.2612
Gammaproteobacteria	Burkholderiaceae	Sphaerotilus	0.80601	0.27312
Deltaproteobacteria	Bdellovibrionaceae	OM27 clade	0.80212	0.13048
Planctomycetacia	Pirellulaceae	Pirellula	0.7768	0.31085
Alphaproteobacteria	Magnetospiraceae	UK_Magnetospiraceae	0.74448	-0.025
Alphaproteobacteria	Sphingomonadaceae	Sphingomonas	0.70377	-0.306
a15:0	a15:0	a15:0	0.69999	0.0721
Bacteroidia	UK_SM1A07	UK_SM1A07	0.69286	-0.394
Gammaproteobacteria	Burkholderiaceae	Mitsuaria	0.66156	-0.1493
Agaricomycetes	UK_Agaricomycetes	UK_Agaricomycetes	0.61129	-0.3267

Table S4. Pearson correlations of OTU results at class, family, and genus levels, assimilation of ^{13}C-polypropylene to phospholipid fatty acids, with NMDS1 and NMDS2 axes.

CLASS	FAMILY	GENUS	NMDS1	NMDS2
Alphaproteobacteria	Elsteraceae	UK_Elsteraceae	0.98891	0.05127
Acidobacteriia	Solibacteraceae (Subgroup 3)	Candidatus Solibacter	0.98278	-0.1223
Deltaproteobacteria	Haliangiaceae	Haliangium	0.97806	-0.1132
Verrucomicrobiae	Pedosphaeraceae	Pedosphaera	0.97791	-0.1445
BrSFA	BrSFA	BrSFA	0.97451	-0.145
Alphaproteobacteria	Devosiaceae	Devosia	0.97306	0.12467
Bacteroidia	Chitinophagaceae	Heliimonas	0.97293	-0.1259
a15:0	a15:0	a15:0	0.9633	0.69685
Gammaproteobacteria	Burkholderiaceae	Leptothrix	0.96263	-0.1103
16:1ω7	16:1ω7	16:1ω7	0.96012	-0.1066
Alphaproteobacteria	Xanthobacteraceae	Pseudolabrys	0.9553	-0.128
Alphaproteobacteria	Acetobacteraceae	Acidisoma	0.95418	-0.1741
Gammaproteobacteria	Burkholderiaceae	CM1G08	0.94802	-0.1821
Chthonomonadetes	Chthonomonadaceae	Chthonomonas	0.94703	-0.1821
Phycisphaerae	Phycisphaeraceae	I-8	0.9462	-0.1741
Gammaproteobacteria	Pseudomonadaceae	Pseudomonas	0.94473	-0.0784
BD7-11	UK_BD7-11	UK_BD7-11	0.94215	-0.2028
18:1ω9	18:1ω9	18:1ω9	0.93778	-0.0653
Monoblepharidomycetes	UK_Monoblepharidomycetes	UK_Monoblepharidomycetes	0.92731	-0.077
Alphaproteobacteria	Acetobacteraceae	Rhodovastum	0.91827	-0.2092
Gammaproteobacteria	Rhodocyclaceae	Zoogloea	0.90832	-0.2723
Deltaproteobacteria	Archangiaceae	Anaeromyxobacter	0.90405	-0.2237
Planctomycetacia	Isosphaeraceae	Singulisphaera	0.89724	-0.208
Babeliae	UBA12409	UK_UBA12409	0.89264	-0.1313
i15:0	i15:0	i15:0	0.87943	0.18482
Fimbriimonadia	Fimbriimonadaceae	UK_Fimbriimonadaceae	0.87781	0.25253
i14:0	i14:0	i14:0	0.86908	-0.2929
Verrucomicrobiae	Pedosphaeraceae	UK_Pedosphaeraceae	0.84323	-0.0519
TK10	UK_TK10	UK_TK10	0.8356	0.36346
Acidobacteriia	Solibacteraceae (Subgroup 3)	Bryobacter	0.8309	0.01499
Deltaproteobacteria	Polyangiaceae	Aetherobacter	0.82127	0.0903
Gammaproteobacteria	Burkholderiaceae	Ideonella	0.81058	0.11486
Chloroflexia	Roseiflexaceae	UK_Roseiflexaceae	0.80809	0.46946
18:1ω7	18:1ω7	18:1ω7	0.80596	-0.1996
Gammaproteobacteria	Burkholderiaceae	Comamonas	0.75885	-0.2578
Gammaproteobacteria	Solimonadaceae	Nevskia	0.68226	0.07603
Deltaproteobacteria	0319-6G20	UK_0319-6G20	0.67652	-0.2356
Malasseziomycetes	Malasseziaceae	Malassezia	0.61552	-0.2731

Table S5. Pearson correlations of OTU results at class, family, and genus levels, assimilation of ^{13}C-polyethylene to phospholipid fatty acids, with NMDS1 and NMDS2 axes.

**CLASS**	**FAMILY**	**GENUS**	**NMDS1**	**NMDS2**
Verrucomicrobiae	Pedosphaeraceae	Pedosphaera	0.99414	-0.0514
Gammaproteobacteria	Solimonadaceae	Nevskia	0.99193	-0.0511
Deltaproteobacteria	Archangiaceae	Anaeromyxobacter	0.99057	-0.0365
BD7-11	UK_BD7-11	UK_BD7-11	0.98997	-0.0111
Alphaproteobacteria	Xanthobacteraceae	Pseudolabrys	0.98907	-0.0658
Planctomycetacia	Pirellulaceae	UK_Pirellulaceae	0.98867	0.00756
Alphaproteobacteria	Elsteraceae	UK_Elsteraceae	0.98718	-0.0993
Alphaproteobacteria	Acetobacteraceae	Acidisoma	0.98692	-0.0608
a15:0	a15:0	a15:0	0.9865	0.26755
Chthonomonadetes	Chthonomonadaceae	Chthonomonas	0.98454	-0.0576
Gammaproteobacteria	Rhodocyclaceae	Zoogloea	0.98385	-0.0503
Acidobacteriia	Solibacteraceae (Subgroup 3)	Candidatus Solibacter	0.98255	-0.0443
Babeliae	UBA12409	UK_UBA12409	0.98223	-0.0687
Acidobacteriia	Solibacteraceae (Subgroup 3)	Bryobacter	0.98217	-0.0614
Phycisphaerae	Phycisphaeraceae	I-8	0.97896	-0.0275
Deltaproteobacteria	Haliangiaceae	Haliangium	0.97639	-0.057
Gammaproteobacteria	Burkholderiaceae	CM1G08	0.97627	-0.0247
Planctomycetacia	Isosphaeraceae	Singulisphaera	0.97237	-0.0422
Bacteroidia	Chitinophagaceae	Heliimonas	0.96307	-0.0639
Alphaproteobacteria	Devosiaceae	Devosia	0.96265	-0.0239
Verrucomicrobiae	Pedosphaeraceae	UK_Pedosphaeraceae	0.95491	0.04939
Gammaproteobacteria	Burkholderiaceae	Duganella	0.95165	0.2793
Gammaproteobacteria	Pseudomonadaceae	Pseudomonas	0.94628	-0.1809
Monoblepharidomycetes	UK_Monoblepharidomycetes	UK_Monoblepharidomycetes	0.93154	-0.0996
Alphaproteobacteria	Rhodobacteraceae	Rhodobacter	0.9102	0.38665
18:1ω9	18:1ω9	18:1ω9	0.90629	-0.136
Alphaproteobacteria	Acetobacteraceae	Rhodovastum	0.87414	0.27561
Chloroflexia	Roseiflexaceae	UK_Roseiflexaceae	0.85439	0.3377
Fimbriimonadia	Fimbriimonadaceae	UK_Fimbriimonadaceae	0.85433	0.29395
BrSFA	BrSFA	BrSFA	0.8526	-0.3045
i14:0	i14:0	i14:0	0.83039	-0.0559
Deltaproteobacteria	Polyangiaceae	Aetherobacter	0.82326	-0.037
18:1ω7	18:1ω7	18:1ω7	0.77044	0.95285
i15:0	i15:0	i15:0	0.73821	-0.1349
TK10	UK_TK10	UK_TK10	0.7085	0.63137
Agaricomycetes	UK_Polyporales	UK_Polyporales	0.69567	-0.0005
Gammaproteobacteria	Burkholderiaceae	Rhodoferax	0.67871	0.63237
Chlamydiae	Simkaniaceae	Rhabdochlamydiaceae bacterium cvE99	0.64107	-0.6491
Agaricomycetes	Stereaceae	Stereum	0.61136	0.25586

SI References

1. Fry B. Stable isotope ecology. New York, NY: Springer; 2006. 308 p.

2. Egli T. Nutrition, Microbial. In: Schaechter M, editor. Encyclopedia of Microbiology (Third Edition) [Internet]. Oxford: Academic Press; 2009 [cited 2022 Mar 15]. p. 308–24. Available from: https://www.sciencedirect.com/science/article/pii/B9780123739445000833

3. van Erven G, de Visser R, Merkx DWH, Strolenberg W, de Gijsel P, Gruppen H, et al. Quantification of Lignin and Its Structural Features in Plant Biomass Using 13C Lignin as Internal Standard for Pyrolysis-GC-SIM-MS. Anal Chem. 2017 Oct 17;89(20):10907–16.
